# Pilonidal sinus disease: Preliminary case-control study on heat-related wound dehiscence

**DOI:** 10.1016/j.amsu.2019.07.032

**Published:** 2019-08-18

**Authors:** Frazzetta Giuseppe, Di Giovanni Silvia, Rosi Patrizia, Pertile Riccardo, Di Sipio Antonio, Rizzo Salvatore Aldo, Inviati Angela, Mascagni Pietro, Mascagni Domenico, Turri Luciano

**Affiliations:** a Azienda USL- IRCCS di Reggio Emilia; b Presidio Ospedaliero San Cimino, General Surgery Department, via S. Cimino, 90018, Termini Imerese, Sicily, Italy; c Ospedale di Trento, Clinical and Evaluation Epidemiology Service, viale Verona, 38123, Trento, Trentino-Alto Adige, Italy; d ASP Trapani, Ospedale SantoVito e Spirito Alcamo, Sicily, Italy; e IRCAD EITS, Place de l'Hôpital, 67000, Strasbourg, France; f Universita degli Studi di Roma La Sapienza Dipartimento di Medicina Clinica, General Surgery, Roma, Lazio, Italy; g Ospedale di Cavalese, General Surgery Unit, Cavalese, Trento, Italy

**Keywords:** Sinus pilonidalis, Wound dehiscence, Heat damage, Pilonidal sinus disease

## Abstract

**Background:**

Pilonidal disease is a morbid condition of the young population, that could impair quality of life with a high cost for the health care system. No consensus exists on optimal surgical treatment, even if several techniques have been proposed. In this preliminary case-control study we compared excision by knife and diathermy to investigate if wound dehiscence could be related to heat spreading during excision of the sinus.

**Materials and method:**

Between January 2017 and February 2018, 29 patients underwent to sinus excision.16 patients underwent sinus excision by diathermy (named “Hot” group, case-group) while 13 patients underwent excision by the knife as the control group (named “Cold” group). The temperature data were recorded for both groups. Were considered primary and secondary outcomes.

**Results:**

the cold group has worse outcomes in operative time and blood loss, but better results in post-operative pain at first day and first control, number of weekly and total dressings until healing, time for full wound recovery, days to return to work, patient feeling feedback and scar aspect. Wounds healed within 8–12 days were 84.6% in the Cold group and 18.8% in the Hot one. I° Dindo-Clavien complications were respectively 15.4% and 100.0% for the Cold and Hot group. No differences were recorded for II° Dindo-Clavien complications and in days of hospitalization.

**Conclusion:**

cold excision of the sinus pilonidalis has better results both in terms of precarious healing and quality of life, probably because the tissues are not subjected to diathermocoagulation damage and therefore the healing occurs more quickly. (United States National Institutes of Health, www.clinicaltrial.gov, number NCT 03764657, www.researchregistry.com UIN 5003).

## Highlights

• Pilonidal sinus disease.
• Cold and Hot surgical procedures.
• Wounds healed within 8–12 days were 84.6% in the Cold group and 18.8% in the Hot one. I° Dindo-Clavien complications were 15.4% for the Cold and 100.0% for the Hot group.

## 1 Introduction

Pilonidal disease is a “minor” but, often potentially invaliding morbid condition that affects a wide slice of young people, lives of whom could be severely impaired in quality. Incidence reaches 0,7% in male patients in a range of age 15–45 years, being exceptional in younger or older ones [[1], [2], [3]]. Considering that in 98.9% the age of affected patients range from <15 to >50, being this a “productive age”, and that, if not well treated, this disease could become a “never-ending story”, clearly that it could have a heavyweight on social and economic aspects. A delay in healing turns in longer hospitalization, high use of painkiller drugs, in several wound controls and lack of days of work.

The impact on the health care system and economic community could be very high. For this reason, a recent renewed interest in the comprehension of arising mechanisms and in finding the ideal method of treatment, lead to the development of a bit of technique and different approaches, from plastic reconstruction to the mininvasive way. We hypothesized this preliminary case-control study comparing excision by knife and diathermy to deepen if the thermal effect of diathermy, may impair wound healing and if wound dehiscence could be related to heat spreading during excision of the sinus. In literature, the reports are few and clinical studies on a comparison between diathermy and classical blade sinus excision are rare.

## 2 Materials and Method

Between January 2017 and February 2018, in our General Surgery Unit, were performed 29 pilonidal sinus excision using knife or diathermy, and primary midline closure, on selected patients, to exclude other risks factors influence that could impair the study. We considered 16 sinus excision by diathermy as a case group (named “Hot” group) and the last 13 procedures performed by knife as control group (named “Cold” group). No Approval of the Ethics committee was required; all patients signed informed consent to the surgical procedure. The work has been reported in line with the STROCSS criteria [17].

### 2.1 Patients selection

All patients were enrolled by outpatient surgical control for signs and symptoms of chronic sinus referred to us by a family doctor; median age was 19.2 ± 4.3 years, ranging from 14 to 52 years old; 6 patients were females and 23 males and them were request to give informed consensus to underwent to pilonidal sinus excision; 16 patients of whom 12 males and 4 females, were treated by electrosurgery as case group (“hot”), while in the control group (“cold”), 13 patients 11 were male and 2 were females. Median time elapsed from first symptoms and surgery was about 1.2 ± 2.3 months. Only two patients had ASA score III, no one of IV ASA score was enrolled and mean pre-operative pain, valued by VAS was 2.5 (±1.5). Inclusion criteria were: male and female patients between 14 and 65 years old, primary, non recurrent pilonidal disease, midline and intergluteal location, spinal anesthesia, dimension no more than 5 cm in length, primary closure performed; Exclusion criteria were the following: age <14 and >65 years old, secondary fistulous tracts or lateral developing/cutaneous opening; dimension over 5 cm in length, local anesthesia employ; smoker and obese patients (BMI> 25 kg/m2), diabetic and coagulopathies affected ones; flogged or acute or infected or abscessed forms, ASA score > IV, normal range WBC and Hb preoperative values. Characteristics and selection criteria are resumed in Table 1.

**Table 1:** Patient's characteristics.

Criteria	Inclusion	Exclusion
Sex	M/F	
Age (year)	14–65	<14 >65
ASA score	I-II-III	IV
BMI	<25 kg/m2	>25 kg/m2
Disease	Primary Not acute	Recurrence Acute/abscess
Localization	Midline	Lateral/perianal
Dimension	<5 cm	<5 cm
Anesthesia	Spinal	Local
Co morbidities and Habits	None	Diabetes/coagulopathies/other/smokers
Blood assessment	Normal	> WBC < Hb

**Fig. 1:**
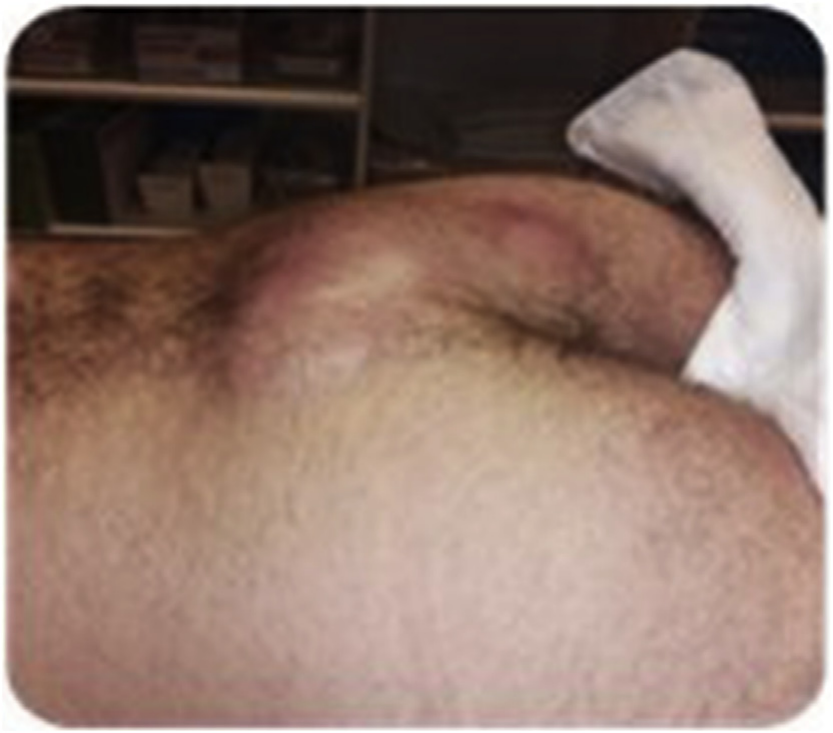
Infected sinus.

**Fig. 2:**
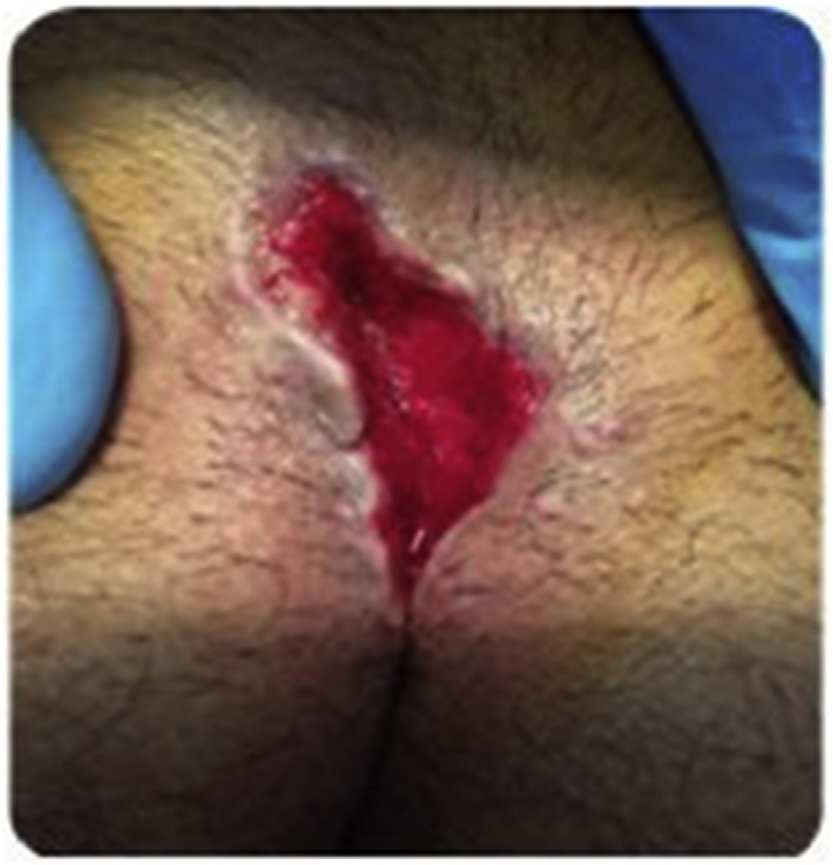
Delayed closure.

In Fig. 1, Fig. 2 we report respectively infected sinus and delayed closure.

### 2.2 Surgical technique

**Fig. 3:**
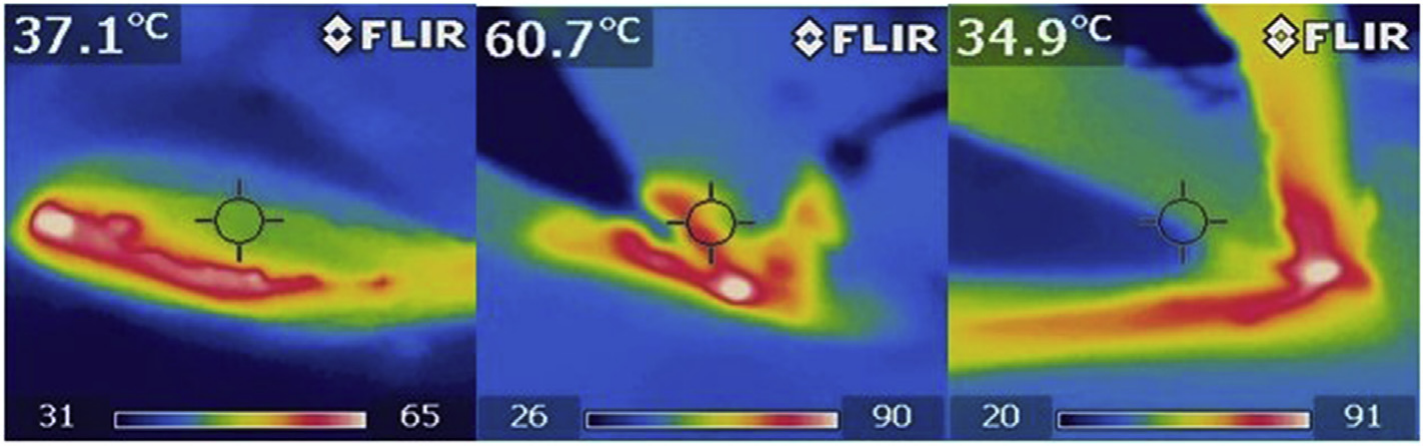
Thermal camera data.

In “hot” group 16 patients were treated by diathermy while in “Cold” group 13 patients were treated by scalpel. A Short Term antibiotic prophylaxis using Cefazoline 2 gr in 100 cc of saline solution was routinely administered and subarachnoid anesthesia employed after bland sedation with Midazolam 2–3 ml i.v. Patients were placed in prone position on operating table with the gluteal line opened wide by adhesive patches; after skin disinfection, dimension and extension of cystic disease was studied by injection of hydrogen peroxide through superficial skin orifice. The same procedure was performed: an elliptical incision on midline around the sinus was made and the sinus was excided laterally and in depth on healthy tissue, down till the pre-sacral fascia: this common approach was made both using scalpel and electrosurgery in the two groups. Monopolar knife, used in hot group, (ForceTriad™ energy platform, Valleylab™ Covidien, Medtronics), was setted for common standard open surgery with forced and spray assessment 35/40 W respectively. During dissection and excision, in each group were measured and recorded the temperatures developing, both on the section surface using an infrared thermometer and thermal imaging camera, and deeper until 1 cm from the section frontline using a “immersion thermometer” sealed by a steri-drape to guarantee the sterility on surgical field (Fig. 3).

We documented, in “off-field testes”, that the isolation of the thermometer by a sterile adhesive film, does not impair and does not alter temperature values recorded, because the difference between recording with and without sterile film was minimal, causing only loss of 0,2-0,5 °C: this could not be relevant. Accurate hemostasis control was obtained using monopolar coagulation in “hot” group one and single stitches in “cold” one; the cavity was washed and irrigated by saline solution and a primary closure was performed using resorbable separated 0/0 stitches. A moderate compression by medical dress completed the procedure. No drains were used. Patients were discharged the day after in the morning or the afternoon and the dress controlled before discharge. Follow-up was planned for interval control until complete healing, and patient were seen after 4, 8, 12 days and then based on wound evolution. A telephone number was given for any problems.

### 2.3 Evaluation of surgical outcomes

We established as primary outcomes, the number of wound completely healed after 8–12 days, the total time, in days, needed to complete wound healing by first-intention midline closure, without delaying from the “standard” way, the number of dressing in week and total ones until complete healing during outpatient management and time needed for hospitalization and to return to work.

Secondary outcomes were valued too, to assess: number and types of complications (Dindo-Clavien), post-operative pain in first postoperative day and at first control by VAS, operative time for complete excision of the sinus, blood loss, patient's feeling feedback, scar aspect at the end of complete healing.

### 2.4 Statistical evaluation

Test F for variances and Fisher exact test were used between two groups.

## 3 Results

Statistical evaluation are reported in Table 2 and Table 3. The comparison between the case group “hot” and case control group “cold” in terms of outcomes, shows that the “cold” has a significantly higher median value compared to the “hot” one referring to the operative time and blood loss. On the other hand, were observed significantly better mean values for the “cold” technique in several outcomes variables: pain after intervention at first day, pain after intervention at first control, number of dressings in week, number of total dressings until recovery, time for a full recovery (days), days for coming at work, patient feeling feedback and scar aspect vas, as showed by P value. Also the comparison on the proportion of recovered wounds after 8–12 days and Dindo-Clavien complications-grade I, shows that the “cold” technique is safer than “hot”, in fact the percentage of wounds healed within 8–12 days is about to 84.6% in the “cold” group compared to 18.8% of the “hot” group. I degree Dindo-Clavien complications recorded about to 15.4% for the “cold” technique and 100.0% for the “hot” one. No significant differences were recorded for II degree Dindo-Clavien complications.

**Table 2:** Selection criteria.

	Cold (n = 13)	Hot (n = 16)	p-value
Time for intervention (minutes)	25.7 (36.4)	13.4 (6.3)	<0.0001
Blood loss (ml)	12.7 (6.7)	5.9 (3.1)	<0.0001
Pain after intervention (first day)	3.3 (0.7)	5.1 (0.7)	<0.0001
Pain after intervention (at first control)	3.3 (1.4)	4.5 (1.0)	<0.01
Number of dressings - week	1.5 (0.4)	3.2 (0.8)	<0.0001
Number of total dressings - until recovery	2.7 (2.7)	7.8 (16.6)	<0.0001
Time for a complete recovery (days)	11.0 (50.3)	28.3 (228.2)	<0.001
Days of hospitalization	1.1 (0.1)	1.3 (0.1)	0.09
Days for coming at work	11.7 (34.1)	28.8 (142.6)	<0.0001
Patient feeling feedback	7.9 (0.6)	5.3 (2.5)	<0.0001
Scar aspect VAS	8.1 (0.3)	5.4 (1.3)	<0.0001

**Table 3:** Temperature data.

	Cold (n = 13)	Hot (n = 16)	p-value
Recovered wounds after 8–12 days	84.6%	18.8%	<0.001
Dindo Clavien complications-grade I	15.4%	100.0%	<0.0001
Dindo Clavien complications-grade II	0.0%	6.3%	0.36

In the group treated by classic scalpel, the number of complete healing in 8–12 days was very high and 11 patients, corresponding to the 84.6%, had a complete resolution; only two patients (15.4%) had delayed healing. Mean time to complete healing, measured in days, was shorter for “cold” group about 8.5 with a range between 6 and 11 days, while longer healing time in the “hot” group with a median of 28.25 in a range of 10–62 days. This was related to the number of dressings in week, about 2 ± 1 in the “cold” group and 3 ± 2 in the “hot” one and also the number of total dressing until healing, very high in some cases, such as 15 changes. Some differences were notice about quality of life and return to work and daily activities: median time to return to normal activity was 25.8 days until 45 days for “hot” group. Time of hospitalization was similar. A better outcomes feedback was recorded also for the “cold” ones, with low incidence of postoperative pain and postoperative complications, reaching 100.0% in “hot” group for Dindo-Clavien Grade I. Blood loss were little higher in the scalpel group, but satisfaction of the patients and cosmetics results were quite high in the cold group too. In this study blood loss was a secondary outcome that we measured using a common suction system connected to a receptacle, with volume marks, to maintain cleaned the operative field. Blood was collected, measured and compared between the two groups. More post-operative pain was recorded at the first control and worse scar aspect at the end of complete healing and also worse feeling feedback was recorded among the patients treated by electro-surgery on VAS evaluation. The average number of days of hospitalization is not significantly different between the two groups.

## 4 Discussion

Several medical and surgical options have been proposed to manage at its best the disease and to reduce recurrence rates and, so, different techniques were introduced with the purpose to improve wound healing and reduce morbidity related. The “ideal surgery” should be simple and short in time, with rapid recovery and short hospitalization, faster return to normal activity, with low recurrence rates, and in end, good cosmetic results. Small incision or limited excision, rather than large removal with delayed closure or flaps creation are some of the very wide approaches to the disease, but until now which should be the gold standard surgery is still a debate [[4], [5], [6], [7]]. Different energy sources have been employed to improve outcomes, but no consensus does exist on real advantages [8,9] and minimally invasive approach seems to be the current trend [[10], [11], [12]]. However no one of these techniques seems to be without postoperative complications as pain, recurrence rates, wound infection and delayed healing. One of the worst aspect in pilonidalis disease management regards exactly wound evolution in post operative time, because a primary closure with a proper healing between 7 and 10 days is the desirable and normal course, but a problem in wound healing should because of weighty morbidity, annoying and limiting consequences on activities of daily living, forcing the patients to several outpatient access and medication until 30–40 days; this clearly could became a stressing situation [13,14]. Secondary intention pilonidalis wound healing, may requires a very long laps of time, from 2 to 6 months or even up to 1–2 years, to return to normality and this traduces in several and repeated medical dressing with patients and hospital burdensomeness [15,16]. Moving by the observation that a cut, an incision or injury heals better and faster if the tissues are healthy, well vascularized and not so much damaged by chemical or physical agents, we hypothesized that, in addition to classical risk factors for recurrence and wound healing failure, an independent technical cause may worsen the clinical course and outcomes delaying healing. This is especially true in burn injuries, were tissue are severely and variably mortified by the heat and the biological recovery is very slow impaired by fluid secretions, exudations, edema, necrotizing areas development or fibrin deposition. We notice in our daily experience in outpatient management, that, often, different course was evident between patients treated by classical diathermy or knife: while a greater incidence of wound complications, specially infection but, above all dehiscence, and delayed healing was noticed in the first ones, on the contrary, a rapid recovery, often, distinguished the second ones.

Aim of this study was to evaluate if energy employed in surgical technique, can improve or worse outcomes. No evaluation was made on recurrence rates and problem related, because we want to deepen what can influence patients’ quality of life. Surely, a great number of outpatient access, several dressing changes, moist sensation and embarrassing feeling related, but also delayed return to normal activity, are very bad aspects of this disease. The purpose of the healing process is to replace the tissue that has been damaged, with living tissue, and to restore the continuity of the skin. Open wounds, heal through a long and complex process by formation of granulation tissue and re-epithelialization, that requires several days, maintaining of good local condition of cleaning and vascularization. If the healing process of a closed wound is “regular”, generally it is completed in 8–10 days without complications and integrity of tissues, from down to up, is replaced. A Such “regular” and fast healing process is the aim of the surgeon and the hope of the patient, but sometimes it is slow and difficult with important delaying tissue sealing due to the creation of unfavorable conditions. Generally a smaller tissue damage and loss, is correlated to a faster and more physiologic healing process; big tissues destruction, damaging and mortification can lead to necrosis, even if partial or isolated, edema, excessive exudation production, chronic inflammatory state, overpressure and tension in the wound with delayed new tissue ingrowths and lack of sealing with dehiscence of sutured skin and subcutaneous tissues. Example of non or delaying healing wounds, where necrosis, exudates, seroma formation, and difficult growth are evident, could be venous legs ulcers, pressure ulcers, skin and soft tissues burns, infected wounds, sutured “no-tension-free” wounds, excessively exuding injury; on the contrary, cutting wound that are not burned or without wide tissue lack or destruction, sutured with no tension among the margins seems to heal faster and better especially if blood or serous secretion are periodically drained. The entity of tissue damage and the nature of the energy and the time of its application on tissues could impair the regeneration events and growth process; in particular thermal energy, as diathermy, used to incision and tissue dissection seems to be associated with more cellular damage and soft tissue devastation. Have been shown faster operating times, reduced blood loss and early post-operative pain and lower analgesia using diathermy for skin incision if compared with cold blade [18,19], but this is a “speedy procedure” for a linear and regular cutting with short and “constant moving” energy application and quickly heat dispersion and rapid return of cut tissues to normal body temperature. In this use the damage and the heat burn is limited to the section line and short in time, with no involvement and spreading to the close tissues. The results could be different when diathermy is used to cut skin, dissect underlying soft tissue and fat and to control bleeding, because this use implies not only a longer the time of contact and of energy application and higher energy concentration in a restricted area for a longer time compared to a linear incision, but also a greater heat development in small areas and a wide spreading in near healthy tissue. The full thickness heat dissection, produced by diathermy excision with cautery, translate in a bigger harm from heat, like in second-third degrees burns producing edema, serous secretion, necrotic areas covered with fibrin film, excess of exudates with high concentration of proteases and reduced level of grown factors, inhibiting cell migration and tissue regeneration, high intra-wound pressure and tension on suture stitches, leading to maceration of the surrounding skin and, in end, as demonstrating by several studies showing that high levels of neutrophil-derived proteases are associated with chronic, non-healing wounds, to an high probability of wound failure [20].

There are few literature reports regarding comparison between different energy source used to perform pilonidal sinus excision. Main data are related only to evaluation of skin incision in animal and abdominal laparotomy in human. No scientific works can be find in literature on comparison between electronic and cold scalpel for the excision of pilonidal disease. First report regarding the use of monopolar electrosurgery for hemostasis in human surgery has been well described since 1926 [21]. Several experimental studies on animals, demonstrated that incisions made by monopolar electrosurgery can lead to a reduction of tensile strength, delayed healing, increased infection and seroma rate and a greater areas of necrosis if compared to scalpel incision [[22], [23], [24], [25]]. Recently, some clinical trials on human tissue have rebutted these conclusions, suggesting that a judicious use of monopolar electrosurgery, can reduced blood loss and surgical time, without influencing inflammation or complications associated with skin incisions [26,27]. This discrepancy may be explained by more aggressive use of coagulation or blended waveforms with higher power settings, leading to excessive collateral tissue damage, as described in older research [28].

Charoenkwan et Al. in a recent Cochrane Database Systematic Review, analysing 1901 participants in nine randomised controlled trials, found no statistically significant difference in overall wound complication rates, nor in rates of wound dehiscence. An update of this research including seven additional RCTs for a total of 16 studies on 2769 participants demonstrated no clear difference in wound infections between electrosurgery and scalpel (7.7% for electrosurgery versus 7.4% for scalpel), no difference in time incision and blood loss; it seems unclear if electrosurgery decreases wound dehiscence compared to scalpel (2.7% for electrosurgery versus 2.4% for scalpel). The Authors concluded that both these comparisons are weak and certainty of evidence was moderate to very low due to risk of bias and imprecise results [29,30]. New “mini invasive” treatments were proposed with the aim to local destruction of tissue so-called pit picking as laser treatment, fistuloscopy and phenol injection; but final evaluation of the results is still pending [[31], [32], [33]]. Petersen in a recent work, analysing the best treatment options, states that maybe tailored approach and patient-oriented planned therapy, can offer better outcomes.

## 5 Conclusions

Data emerging from this preliminary study, seems to suggest that excision by classic blade is safer and superior than using diathermy and seems to have a better impact on patient's postoperative quality of life than the excision using electro-surgery, improving and speeding up surgical recovery. Maybe the choice should be evaluated on the basis of sinus characteristics, patients characteristics, risks factors, personal and work habits, anatomical conditions, family history, patients compliance and collaboration; however seem from these preliminary results that “less damage” are related with “best healing” supporting “non-energy, non-hot” excision of the sinus.

Because this condition is generally a benign disease, often small, in a unfavorable place and that could became very uncomfortable for the patient, it is worth trying for better and less.

This is a preliminary study with the limits of a small sample and short follow-up, but will be extended to other hospitals to carry out a multicenter randomized controlled trial involving a large number of patients and will be completed with anatomopathological analysis of the percentage of necrosis areas and with the analysis of costs for the health care system.

## Ethical approval

No Approval of the Ethics committee was required; all patients signed informed consent to the surgical procedure.

## Unique identifying number (UIN)

NCT 03764657.

https://clinicaltrials.gov/ct2/

## Sources of funding

None.

## Author contribution

Frazzetta, Giuseppe: study design, data collections, data analysis, writing.

Di Giovanni, Silvia: data collections and writing.

Rosi, Patrizia: data analysis.

Pertile, Riccardo: data collections and data analysis.

Di Sipio Antonio: data collections.

Rizzo, Salvatore Aldo: writing.

Inviati, Angela: study design, data collections.

Mascagni, Pietro: Supervision, validation;

Mascagni, Domenico: data analysis.

Turri, Luciano: study design, data collections.

## Conflicts of interest

None.

## Trial registry number

None.

## Guarantor

The Guarantors are Frazzetta Giuseppe, Di Giovanni Silvia, Inviati Angela.

## Provenance and peer review

Not commissioned, externally peer reviewed.
